# The Effect of Code-Switching Experience on the Neural Response Elicited to a Sentential Code Switch

**DOI:** 10.3390/languages7030178

**Published:** 2022-07-11

**Authors:** Angélique M. Blackburn, Nicole Y. Y. Wicha

**Affiliations:** 1 Department of Psychology & Communication, Texas A&M International University, Laredo, TX 78041, USA; 2 Department of Neuroscience, Developmental and Regenerative Biology, The University of Texas at San Antonio, San Antonio, TX 78249, USA

**Keywords:** bilingualism, bilingual comprehension, code switching, code-switching positivity, event related potentials, N400, LAN, LPC, semantic processing, sentence reading

## Abstract

Switching between languages, or codeswitching, is a cognitive ability that multilinguals can perform with ease. This study investigates whether codeswitching during sentence reading affects early access to meaning, as indexed by the robust brain response called the N400. We hypothesize that the brain prioritizes the meaning of the word during comprehension with codeswitching costs emerging at a different stage of processing. Event-related potentials (ERPs) were recorded while Spanish–English balanced bilinguals (*n* = 24) read Spanish sentences containing a target noun that could create a semantic violation, codeswitch or both. Self-reported frequency of daily codeswitching was used as a regressor to determine if the cost of reading a switch is modulated by codeswitching experience. A robust N400 to semantic violations was followed by a late positive component (LPC). Codeswitches modulated the left anterior negativity (LAN) and LPC, but not the N400, with codeswitched semantic violations resulting in a sub-additive interaction. Codeswitching experience modulated the LPC, but not the N400. The results suggest that early access to semantic memory during comprehension happens independent of the language in which the words are presented. Codeswitching affects a separate stage of comprehension with switching experience modulating the brain’s response to experiencing a language switch.

## 1. Introduction

Codeswitching—the act of switching between two languages—is a pervasive phenomenon amongst bilinguals around the world. Bilinguals produce codeswitches for a variety of reasons, from emotional expression to linguistic need (for reviews, see Appel and Muysken 2005; Dewaele 2004; Gonzalez-Reigosa 1973; Gumperz and Hernandez-Chavez 1972; Harris 2004; Marian and Kaushanskaya 2008; Myers-Scotton 1993; Pavlenko 2005, 2008). Importantly, in the communicative exchange, although there are certain constraints around which words can be switched (Dussias 2001; Vaughan-Evans et al. 2020), when a switch will occur is relatively unpredictable for the interlocutor. Nevertheless, in order to comprehend the utterance, they must keep pace by quickly and efficiently integrating the codeswitched word into the ongoing sentence.

Comprehending a codeswitch can incur a cost in the form of a delay in processing. For example, bilinguals read passages more slowly when they contain codeswitches than when they do not, both when reading aloud (Kolers 1966) or reading silently (Macnamara and Kushnir 1971). However, the neurolinguistic process that contributes to this codeswitching cost is just beginning to be understood. This study specifically addresses the interaction between codeswitching and processing words for meaning within a greater sentence context. We have taken advantage of the precise temporal resolution of event related potentials (ERP) to measure the timing and type of effect that switching has on accessing word meaning in the bilingual brain.

Reading a codeswitched word in a sentence context reliably elicits a modulation of two ERP components, a left lateralized negativity (LAN) followed by a late positive component (LPC) (Kutas et al. 2009; Beatty-Martínez et al. 2018; Treffers-Daller et al. 2021; Van Hell et al. 2018; Van Hell et al. 2015). These ERP components are thought to reflect working memory (as per Kluender and Kutas 1993) or syntactic analysis (Friederici 1995; Vaughan-Evans et al. 2020) and sentence integration processing costs, respectively (Friederici 1995; Salmon and Pratt 2002; Leckey and Federmeier 2020; Kuperberg 2007; Van Petten and Luka 2012; Brouwer et al. 2017). Both the LAN and the LPC are larger in amplitude when encountering a codeswitch than when reading a word in the same language as the ongoing sentence. In contrast, processing the meaning of words modulates a different component, called the N400, which is a centro-parietal negativity that peaks around 400ms after the onset of any meaningful or potentially meaningful stimulus (Kutas and Hillyard 1980a, 1980b). It is typically larger in amplitude for words that are not supported by the context compared to more predictable words, and can be thought of as an index of the current state of semantic memory (for a review see Kutas and Federmeier 2011). It has been proposed that codeswitches influence the integration of a word into the building sentence context, as well as recognition of the word (Grainger and Beauvillain 1987, 1988; Grainger and O’regan 1992), but there is mixed evidence as to whether codeswitching can modulate the N400.

In their seminal study, Moreno et al. (2002) tested whether the cost of reading a codeswitch occurs at initial access to meaning (e.g., lexico-semantics), or at later stages in comprehension (e.g., integration of the word into the sentence context). ERPs were recorded while Spanish–English bilinguals read simple English sentences (e.g., “The driver of the speeding car was given a …”) that could end with an expected word (ticket), the Spanish translation of the expected word (*multa*), or a plausible English lexical switch (citation). Moreno et al. hypothesized that if codeswitches cause difficulty at the level of processing meaning, reading a codeswitched word would elicit a modulation of N400 amplitude. However, codeswitched words did not modulate the N400. Instead, translations of the expected words (i.e., codeswitches) elicited a negativity with a left anterior distribution characteristic of a LAN. The LAN was followed by a positive-going increase in amplitude between 450–850 ms after stimulus onset, a classic LPC, reflecting the processing cost of the codeswitch. In contrast, lexical switches in the same language elicited a typical N400 modulation over central-parietal electrodes, with larger amplitude for these less expected words compared to the expected continuation.

Other ERP studies of single-word (Ng et al. 2014) and multiple-word (Litcofsky and Van Hell 2017) intra-sentential codeswitching have shown a similar pattern, with a LAN, followed by a LPC (although the LAN is sometimes interpreted as a different component; (Ruigendijk et al. 2016). Notably, the LPC is only present for codeswitches in a sentence context, providing support that the LPC reflects a general processing cost for integrating a switched word into the ongoing sentence context. The LAN appears for isolated words as well as sentences (Molinaro et al. 2011) and might even appear in response to codeswitches in monolinguals (Ruigendijk et al. 2016), reflecting a process that is not specific to comprehending the word. Notably, neither the LAN nor the LPC to a codeswitch appear to be modulated by the amount of context preceding it, such as words appearing earlier or later in a story (Ng et al. 2014). This further suggests that this LAN/LPC complex in response to codeswitches is not sensitive to lexico-semantic information.

There is evidence, however, that these components, namely the LPC, can be sensitive to bilingual specific factors, such as the direction in which the switch occurs (Litcofsky and Van Hell 2017; Fernandez et al. 2019; Liao and Chan 2016), the frequency with which bilinguals experience language switches in daily life (Gosselin and Sabourin 2021; Valdés Kroff et al. 2020), and even the presence of another bilingual during comprehension (Kaan et al. 2020). Moreover, individual variability, such as differences in language proficiency, modulate the codeswitching late positivity (Moreno et al. 2002; Van Der Meij et al. 2011). For example, the more proficient bilinguals are in a language, the smaller the LPC to switches into that language (Ruigendijk et al. 2016; but see Moreno et al. 2002; Van Der Meij et al. 2011). Similarly, the LPC can be modulated asymmetrically depending on the direction of the switch, typically with an increased LPC only when switching into a weaker language (Litcofsky and Van Hell 2017; Fernandez et al. 2019), although the LPC has also been observed in both directions of switch in proficient bilinguals (Liao and Chan 2016), or only when switching into a dominant language (Van Der Meij et al. 2011). The LPC is more robust for codeswitches on nouns than verbs (Ng et al. 2014), is observed even to switched function words (Kaan et al. 2020; Gosselin and Sabourin 2021) and may depend on syntactic structure of the sentence (matrix) language (Vaughan-Evans et al. 2020). Given these findings, the LPC likely reflects processes related to sentence comprehension, more broadly, rather than codeswitching, per se (Yacovone et al. 2021). In turn, we might expect factors such as frequency of experiencing a codeswitch to affect the cost of integrating a codeswitch into the ongoing sentence, as measured by the LPC.

Based on their findings, Moreno et al. (2002) concluded that codeswitches had little to no effect on processing the meaning of an expected continuation (see also review by Moreno et al. 2008). Their N400 results provide strong evidence that sentence context facilitates access not only to related words in the same language, but to the equivalent translations in another language. This is in line with theoretical models of bilingual lexical access that allow for both within- and between-language spread of activation in memory, such as the BIA+ model (Dijkstra and Van Heuven 2002). This is also in line with research suggesting that bilinguals prioritize processing the meaning of words independent of the language in which they are presented (Ng and Wicha 2013).

However, given that Moreno et al. (2002) only used translations of the expected continuation, which were supported by the sentence context, it is less clear if processing of an unexpected word would be equally unaffected by a language switch, e.g., how far does cross-linguistic activation spread. Several studies in the two decades since this seminal study have provided mixed evidence to suggest that codeswitching might actually incur an additional cost in accessing a word from semantic memory under certain circumstances (Proverbio et al. 2004; Gosselin and Sabourin 2021; Liao and Chan 2016; Yacovone et al. 2021; Van Der Meij et al. 2011; Litcofsky and Van Hell 2017; Valdés Kroff et al. 2020; Ruigendijk et al. 2016).

Unlike Moreno et al. (2002), Proverbio et al. (2004) and Van Der Meij et al. (2011) both reported N400 modulations to a codeswitch. However, in both cases there were methodological choices that might explain the modulation. Proverbio et al. (2004) observed overall larger N400 amplitude for sentence final codeswitches (referred to as mixed sentences) in multilingual professional interpreters. This was a main effect collapsed across sensible and senseless (semantically incongruous) sentences. Interestingly, the lack of an interaction between congruity and codeswitching indicates that codeswitching did not additionally modulate the N400 to senseless words. The items were blocked, such that all sentences either contained a codeswitch or did not, and sentences (not just the final word) appeared in both English and Italian in a single block. The significant differences in the language switching experience of the population (professional interpreters being experienced switchers) and methodology make it difficult to compare their findings to other studies (Moreno et al. 2008). Nevertheless, this study introduced the possibility that codeswitching can affect semantic level processing.

Van Der Meij et al. (2011) also reported an N400 modulation in response to codeswitches in late learners of English (L2) reading English sentences with occasional Spanish (L1) switches (cf. Litcofsky and Van Hell 2017). This N400 increase was small, perhaps because the switched words were adjectives rather than the typical nouns, and was larger for learners with greater proficiency in English. They interpreted the code-switch effect on the N400 as activation cost of lexical forms in the less active language. However, a comparison of late learners to early bilinguals should be interpreted with caution, given that the N400 could hypothetically be specific to learners (e.g., McLaughlin et al. 2010). The LPC to codeswitches was also larger in amplitude for higher compared to lower proficiency individuals and was interpreted as updating the context in light of an unexpected event. High proficiency bilinguals also generated a LAN (similar to Moreno et al. 2002), which the authors argued might be related to integrating the grammatical rules of the two languages. However, the results of the LPC and LAN are inconsistent with later studies which show larger effects when switching into the less dominant language (Fernandez et al. 2019; Litcofsky and Van Hell 2017).

As for whether switches can affect semantic level processes, as indexed by the N400, a handful of recent studies has provided some clues. The presence of an N400 modulation in response to a codeswitch may depend on the direction of the switch, with reports of an N400 amplitude modulation only when switching into a less dominant language (Liao and Chan 2016). This suggests that lexical-semantic costs from a switch occur when there is less proficiency in accessing information from that language. Similarly, the frequency of switching may also modulate N400 amplitude with some evidence for the presence of an N400 only for less habitual switchers, although even habitual switchers can show an N400 amplitude modulation for codeswitches when listening to sentences (although not when reading; Fernandez et al. 2019; Litcofsky and Van Hell 2017). Switches into a language that is less frequently used for reading might also decrease the distribution of the N400 effect, with less frontal negativity than non-switches, which may reflect a change in frontal ERP effects related to integration (Ng et al. 2014).

The current study takes another look^1^ at the impact of codeswitching on accessing the meaning of words in a sentence, as indexed by the N400 response, as well as how the frequency of switching in daily life affects all ERP responses to a switch. Early balanced Spanish–English bilinguals read Spanish sentences for comprehension while scalp ERPs were recorded. One noun in each sentence could appear in Spanish or English (codeswitch), and either be semantically congruent or incongruent with the preceding context (see example below). We controlled for some of the potential methodological confounds in the previous studies. Namely, in our study, the same stimuli appeared in all 4 conditions, all 4 conditions appeared mixed (rather than blocked by condition), and the position of the target in the sentence was unpredictable and never sentence final. Using a fully crossed design, which manipulates semantic congruity and the occurrence of a codeswitch on the same word, allowed us to isolate the ERP components elicited by each process and determine at what stages in comprehension, if any, these processes interact.

Two recent studies measured the effect of codeswitching on processing nouns with high or low expectancy within a sentence context (Yacovone et al. 2021; Valdés Kroff et al. 2020). Yacovone et al. (2021) showed an N400 modulation for codeswitches into the less dominant language that was equivalent to the N400 modulation for low expectancy words. However, codeswitches did not additionally modulate the N400 for unexpected words (i.e., no interaction with semantic processing). In contrast, Valdés Kroff et al. (2020) showed no effect of codeswitching on the N400 in proficient early bilinguals, only revealing the typical effect of expectancy on the N400 with smaller amplitude for nouns supported by context. Given that the current study tests proficient early bilinguals, it is possible that codeswitches may not affect lexico-semantic processing, as in Valdés Valdés Kroff et al. (2020). However, in both studies the unexpected nouns were plausible continuations. Here, we determine if codeswitching affects semantic processing of nouns that are plausible, where sentence context supports the interpretation, and implausible, where sentence context does not support an interpretation, in sentences with moderate to high sentence constraint.

A secondary goal of the current study is to determine the extent to which codeswitching in daily life affects comprehension of a switched word. In both of the studies just mentioned, the bilinguals self-reported as habitual switchers (e.g., frequently switched between languages in daily life), and frequency of switching within these frequent switchers only modulated an early positivity, possibly related to target categorization (e.g., the P300, see Valdés Kroff et al. 2020). The frequency with which a bilingual habitually codeswitches has been suggested to modulate the language-control needed to switch between languages when speaking (Blackburn 2013, 2017; Green and Abutalebi 2013; Green and Wei 2014; Kang et al. 2015; Morales et al. 2015; Wu and Thierry 2013), and even alter brain networks involved in switching between other types of tasks (see Blackburn 2019 for a more in depth discussion of the neural impact of language environment; cf. Hernández et al. 2013; Yang et al. 2016; Yim and Bialystok 2012). A number of studies have found that daily codeswitching habits modulate the switching costs incurred during language production (e.g., Kheder and Kaan 2016; Soveri et al. 2011). In particular, the cost of producing a code-switch, operationalized as the difference in response time between switched and non-switched picture naming trials, is decreased in individuals who codeswitch often in their daily lives (Prior and Gollan 2011).

Codeswitching habits appear to affect not only production, but also comprehension of a codeswitch. For example, compared to bilinguals who do not switch frequently, spoken language translators show no behavioral switch cost when reading sentences with a codeswitch into their first language (Ibáñez et al. 2010). In addition, bilinguals who frequently codeswitch become sensitive to specific code-switching patterns (Valdés Kroff et al. 2016, 2017). These studies imply that people who switch often produce and comprehend switches more easily than bilinguals who do not habitually codeswitch (see Blackburn 2018 for a review).

The effect of switching on the LPC, however, is less clear. There is some evidence that expertise, such as in playing chess, leads to larger LPC amplitude, more broadly, as a reflection of improved processing (Wright et al. 2013). Similarly, Gosselin et al. reported an LPC modulation to codeswitches only for more frequent switchers. This is opposite the finding that codeswitching elicits a N400 and LAN only in individuals who switch less frequently (Gosselin and Sabourin 2021), although the authors suggested that the LPC may have also occurred in non-habitual switchers but was obscured by the overlapping N400. In brief, the effect of switching habits has been reported rarely and inconsistently across these studies.

Here, we tested whether codeswitching frequency modulates the amplitude of the LPC, or the earlier negativities, LAN and N400, using a survey developed to measure codeswitching habits. We used the Assessment of Code Switching Experience Survey (ACSES) (Blackburn 2013) to measure how often each participant codeswitches in their daily life. Codeswitching frequency was then correlated with the amplitude of the ERP effects. In the current study, semantic violations were expected to elicit a classic N400 congruity effect and codeswitches were expected to elicit a LAN and LPC. The critical questions are (1) whether codeswitching alone elicits an N400 modulation, as in Yacovone et al. (2021), (2) whether codeswitches additionally modulate the N400 congruity effect or if processing the meaning of the word takes priority, as in Ng and Wicha (2013), and (3) whether switching habits would modulate any of the stages of processing indexed by the LAN, N400 and LPC in early balanced bilinguals.

## 2. Materials and Methods

### 2.1. Participants

Twenty-four (9 female, 15 male) healthy right-handed Spanish–English balanced bilinguals were included in the analysis. Data from one additional participant was excluded due to poor performance and excessive EEG artifacts. Participants were on average 21.5 years old, ranging from 19 to 29 years (SD = 2.3). Participants completed a series of language measures before the ERP recording, including a comprehensive Language History Questionnaire adapted from the LEAP-Q (Marian et al. 2007), the Assessment of Code Switching Experience Survey (ACSES) (Blackburn 2013), and the Woodcock Passage Comprehension test in Spanish and English (Woodcock 1991). All participants reported Spanish as their native language. Participants had an average score of 2.86 (SD = 1.22, range = 1–5.37) for ‘frequency of code-switching’ based on the ACSES, where 1 was “never switches” and 7 was “always switches”. The age of acquisition (AOA) for English was on average 8.08 years, ranging from 0 to 19 years. Spanish and English daily use was comparable; participants reported speaking English 51.25% (SD = 20.76%) and Spanish 48.75% (SD = 20.76) on an average day [paired-samples comparing English and Spanish use *t*_23_ = 0.30, *p* = 0.771).

Balanced bilinguals in this study were defined as those with Spanish and English scores on the Woodcock Passage Comprehension test within one Cognitive-Academic Language Proficiency (CALP) level of each other (Woodcock 1991). The average Relative Mastery Index (RMI) score, on a scale of 0–100 proficiency, was 80/90 for English (SD = 11.1, range = 55/90–97/90) and 82/90 for Spanish (SD = 14.6, range = 45/90–98/90). A paired-samples t-test revealed no significant difference in the RMI scores between the two languages (*t*_23_ = 2.07, *p* = 0.220), indicating that the participants were balanced in proficiency.

### 2.2. Stimuli and Procedure

Materials were 128 sentence pairs in Spanish adapted from Wicha et al. (2004), each containing a target noun of medium to high cloze probability ranging between 50–100% with a mean of 81% (previously normed by a separate sample of native Spanish speakers). The targets were singular, non-cognate nouns. They were never sentence final and could appear anywhere in either the first or second sentence of the pair. The target noun in each sentence pair could be either the expected Spanish noun based on sentence context or not (semantically congruent/incongruent), or the English translation of these. This created four conditions as follows:

#### Control Sentence:

*Caperucita Roja llevaba la comida para su abuela en una* ***canasta*** *muy bonita. Pero el lobo llegó antes que ella*. [Little Red Riding Hood carried the food to her grandmother in a pretty **basket**. But the wolf arrived before her.]

#### Codeswitch:

*Caperucita Roja llevaba la comida para su abuela en una* ***basket*** *muy bonita. Pero el lobo llegó antes que ella*.

#### Semantic Violation:

*Caperucita Roja llevaba la comida para su abuela en una* ***manzana [apple]*** *muy bonita. Pero el lobo llegó antes que ella*.

#### Code Switch–Semantic Violation:

*Caperucita Roja llevaba la comida para su abuela en una* ***apple*** *muy bonita. Pero el lobo llegó antes que ella*.

Every sentence pair and target appeared in all 4 conditions across 4 lists, so that participants saw only one version of each stimulus. Each list contained 32 sentence pairs per condition. Target nouns were first grouped to match grammatical gender, animacy, word length, and frequency (as closely as possible). Word frequencies were obtained from Davis and Perea (2005) (based on the LEXESP database) for Spanish, and from Davis (2005) (based on the CELEX database) for English. The target word for each sentence pair was interchanged with another sentence pair in its group, so that the target for one sentence became the semantic violation for the next. The sentences were then normed for acceptability by 15 native Spanish speakers who did not take part in the ERP study (8 males, 7 females; ages 19 to 39, mean 25 years, SD = 5.55; paid to participate). Participants in the norming study had at least 5th grade proficiency in Spanish on the Woodcock Passage Comprehension test (62.79 to 100% correct, M = 80.90%, SD = 10.12; RMI M = 69/90, SD = 21.12, Range = 23/90–99/90) (Woodcock 1991). Participants in the norming study read all 128 experimental sentences containing a semantic violation in Spanish (no codeswitch), up to and including the target word, as well as 50 filler sentences that did not contain violations and a potential sentence to substitute for the experimental set, if needed. They were asked to rate the sentence for plausibility on a scale of 1–5, where 5 indicates that the sentence made perfect sense and 1 indicates that the target word in the sentence did not make sense. Sentences with violations were rated between 1.4 and 3.1 (M = 2.0, SD = 0.39) and filler sentences without violations were rated between 4.1 and 5.0 (M = 4.79, SD = 0.23), indicating that the sentences containing violations were perceived as implausible continuations of the sentence.

The ERP recording took place in a single session in an electrically shielded, sound-attenuating chamber. Participants were seated approximately 19 inches from a 19-inch CRT monitor. The task was to read the sentences silently for comprehension. Participants were given instructions and a short practice with a token from each stimulus condition. Twenty-four trials (randomly chosen) were followed by a probe sentence in Spanish related to the sentence pair just presented. Participants made a true-false judgment by pressing a button on a keyboard for these probe sentences only. An equal number of probes occurred in each condition, and in the first or second sentence in the pair. The probe sentences were designed to be moderately difficult and were used to ensure attentiveness to the task and sentence content.

The target noun appeared in the first sentence of the pair on 66 trials (early) and in the second sentence on 62 trials (late). To help participants plan eye blinks (to avoid eye movement artifact during the targets), the trials were blocked by whether the target appeared early or late, then randomized within a block. The order of presentation was counterbalanced so that half of the participants began with the early target sentence pairs and half with the late target sentence pairs. The sentence containing the target word was presented one word at a time (300 ms with 200 ms ISI) in the center of the screen, preceded by a 1-s fixation cross (200 ms ISI). The last word of the sentence was followed by a 700 ms ISI before the onset of the next sentence pair. Stimuli were presented as white text on a black background in the Verdana font (letter matrix = 30 by 68 pixels). Participants were asked to minimize blinking during the target sentence and allowed to blink naturally during the other sentence, which was presented as a whole and stayed on the screen until the participant pressed a button to advance.

### 2.3. ERP Parameters

Continuous electroencephalogram (EEG) was recorded from 32 tin electrodes: 26 embedded in a standard cap (Electro-Cap Inc., Eaton, OH, USA) in geodesic array (as per M. Kutas lab convention), and 6 free electrodes to record eye artifacts and mastoid process references. Blinks were monitored using electrodes placed under each eye, and horizontal eye movements through a bipolar recording from the outer canthi of each eye. The data was recorded using an SAI analog bio-amplifier with a band pass filter set from 0.01 to 100 Hz. Electrode impedances were maintained below 5 kΩ. Data was recorded to a left mastoid reference on-line and re-referenced to the algebraic sum of the left and right mastoids (Wicha et al. 2004). The output of the bio-amplifier was fed into a 32 channel 12-bit analogue-to-digital converter and sampled at 250 Hz. ERPSYSTEM software (developed by P. J. Holcomb, Boston, MA, USA) was used to simultaneously present visual stimuli to the participant and deliver event and timing codes to the data acquisition PC at the onset of each word.

Trials with artifacts due to eye movements, excessive muscle activity, or amplifier saturation were eliminated offline before averaging using uniformly applied algorithms. Approximately 8.71% of the total trials (range = 1.5–14.5%) were rejected, with roughly equal loss of data across conditions. Data was averaged for each experimental condition from the onset of the target word, relative to a 100 ms pre-stimulus baseline. A digital band-pass filter set from 0.1 to 30 Hz was used prior to running analyses to reduce EEG content irrelevant to the components of interest.

## 3. Results

One participant was eliminated due to poor performance on the probe questions (58.3% accuracy). Accuracy for the other 24 participants on judging the truth of the sentence probes was high (mean = 85.5%, range = 70.8–95.8%), indicating good comprehension of the sentences and attentiveness to the task.

**Figure 1.**
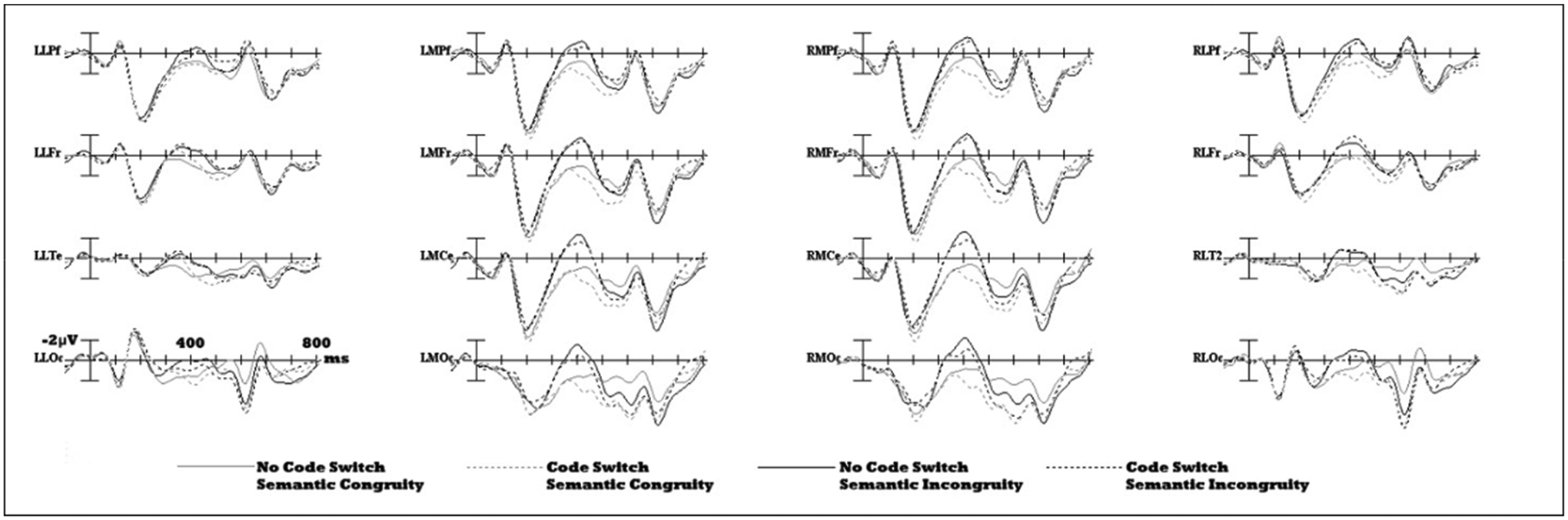
ERP waveforms. Raw waveforms are presented for each condition, time-locked to the onset of each stimulus.

Figure 1 shows grand average ERPs (*n* = 24) to the target noun for each of the 4 conditions: control, semantic incongruity, codeswitch, and codeswitched semantic incongruity. Visual inspection of the waveform reveals equivalent early sensory components across the conditions—P1-N1-P2—followed by slow wave components. Overall, semantically incongruous nouns elicited an enhanced negativity between 300 and 500 ms post-stimulus onset (N400) relative to congruous nouns. Peak latency analysis revealed that the N400 peaked at 399 ms (SD = 28.09 ms) post stimulus onset.

Visual inspection indicates that there may be two distinct but overlapping positivities, a codeswitching positivity which onsets earlier than an LPC to semantic violations. Relative to non-switched Spanish nouns, codeswitched English nouns elicited an enhanced positivity between 320 to 650 ms post-stimulus onset, which overlapped with the N400. Semantic incongruities also elicited a late positive component from 500–800 ms post-stimulus onset. Therefore, an onset latency analysis was performed to obtain reliable onset latencies for the codeswitch positivity and the later semantic violation positivity.

**Figure 2.**
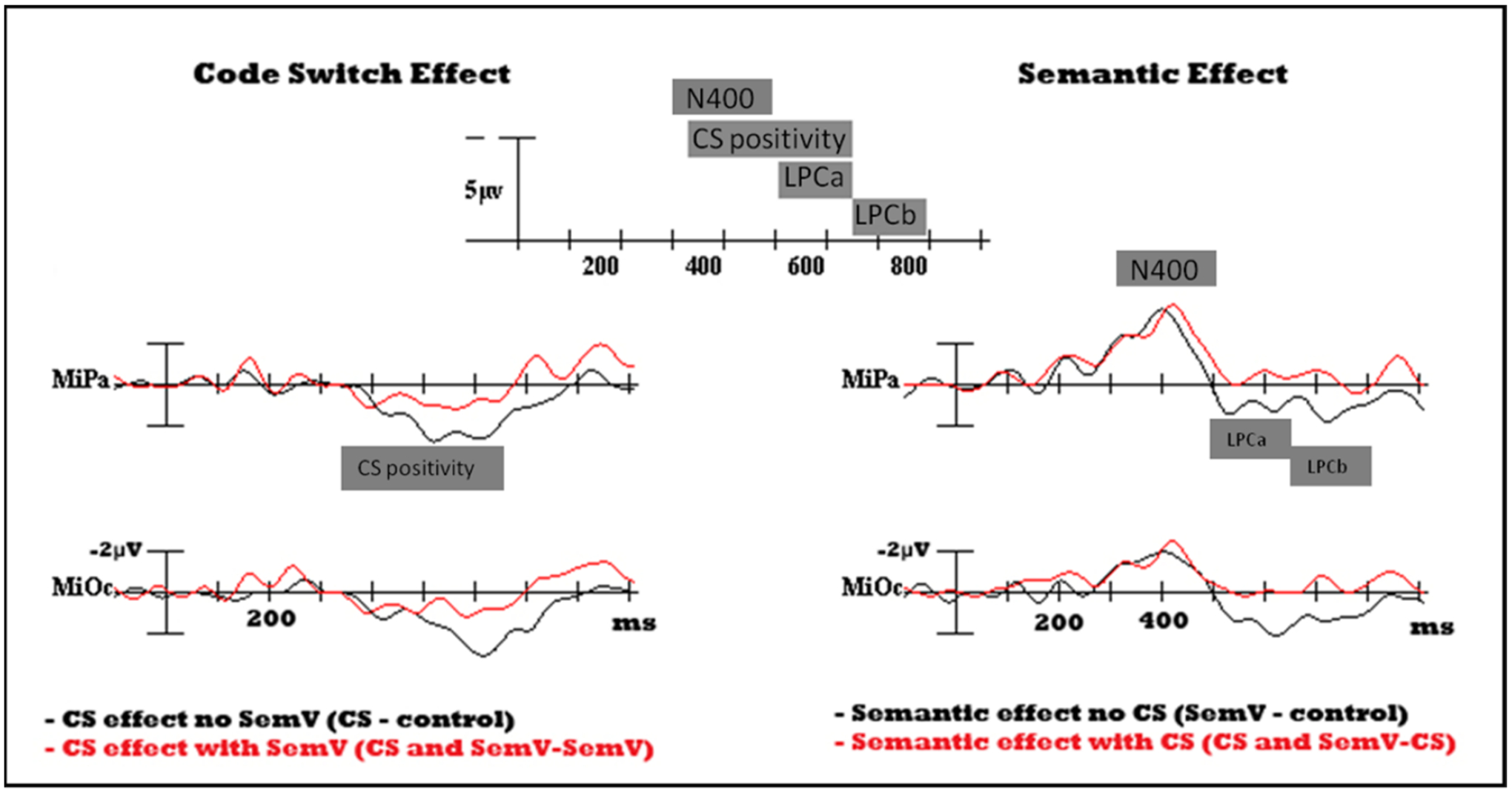
Difference waves of the codeswitch and semantic effects. Difference waves for the codeswitch effect without a semantic violation represent a point-by-point subtraction of the grand averaged response to a codeswitch (CS) minus the control; difference waves for the codeswitch effect with a semantic violation represent a point-by-point subtraction of the grand averaged response to a codeswitched semantic violation (CS and SemV) minus the semantic violation alone (SemV). Difference waves for the semantic effect without a codeswitch are calculated as the response to a semantic violations (SemV) minus the control; the semantic effect with a codeswitch is calculated as the response to a codeswitched semantic violation (CS and SemV) minus the response elicited to codeswitches alone (CS). These waves indicate that code-switches elicit a positivity with a latency onset around 320 ms. This onset overlaps with the first half of the LPC (LPCa) elicited to a semantic violation, but not the LPCb.

Difference waves were obtained for the Code Switch Effect in the absence (codeswitch minus control) and presence of a semantic incongruity (codeswitched semantic incongruity minus semantic incongruity with no codeswitch) [Figure 2]. Likewise, the Semantic Congruity Effect was calculated as a difference wave in the absence (semantic incongruity minus control) and presence of a codeswitch (codeswitched semantic incongruity minus codeswitch without a semantic incongruity). The onset latency was calculated as the time-point of the first positive deflection exceeding 100 ms in duration based on the difference waves. The onset of the codeswitch effect (codeswitches minus non-switches) was similar in the presence (324 ms) and absence (319 ms) of a semantic incongruity, and overlapped in time with the N400. The LPC onset to a semantic incongruity was later than that of a codeswitch and was similar in the absence (447 ms) and presence (424 ms)^2^ of a codeswitch.

Time windows for subsequent analysis were selected based on latency onsets. Due to overlap between the LPC and the codeswitching positivity, the LPC was analyzed in two separate windows, the LPCa from 500–650 ms and the LPCb from 650–800 ms (see Barber and Carreiras (2005) for a similar analysis of the LPC, i.e., P600a/P600b). Mean amplitude was therefore, measured in three non-overlapping time windows—300 to 500 ms (N400), 500 to 650 (overlap between the codeswitching positivity and the semantic positivity^3^—the LPCa), and 650 to 800 ms (LPCb), plus a non-orthogonal fourth window encompassing the entire code-switching positivity (320–650 ms) for comparison of the codeswitching effect to other studies in the literature.

Mean amplitude in each time window was subjected to omnibus repeated-measures ANOVAs with 2 levels of Semantic Congruity (congruous, incongruous), 2 levels of Code Switching (no switch, codeswitch) and 26 levels of Electrode. Interactions by Electrode were further analyzed using a 16-electrode subset with 2 levels of Semantic Congruity, 2 levels of Code Switching, 2 levels of Hemisphere (right, left), 2 levels of Laterality (medial, lateral), and 4 levels of Anteriority (prefrontal, frontal, central, and occipital) to determine the distribution of the effects (Wicha et al. 2004). Pairwise comparisons to explain interaction effects were conducted when appropriate. Regions of interest were selected based on the distributional analyses in order to measure localized effects. Effects were considered significant at *p* < 0.05, with repeated measures with greater than one degree of freedom reported after Greenhouse-Geisser correction.

### 3.1. N400

A main effect of Semantic Congruity, where responses to semantic incongruities were more negative than congruities, was observed [F_(1,23)_ = 79.791, *p* < 0.001] between 300 to 500 ms. The significant interaction between Electrode and Semantic Congruity [F_(2.9,66.6)_ = 9.574, *p* < 0.001] was further analyzed for distributional differences (main effect of Electrode, [F_(4.4,100.7)_ = 4.342, *p* = 0.002]). The distributional analysis revealed interactions of Semantics and Laterality [F_(1,23)_ = 60.793, *p* < 0.001], Semantics, Laterality and Hemisphere [F_(1,23)_ = 6.339, *p* = 0.019], and Semantics, Laterality and Anteriority [F_(2.3,52.1)_ = 7.365, *p* = 0.001], indicating that the effect of Semantic Congruity was widely distributed across the scalp and maximal over right medio-central sites, consistent with the N400 literature. Visual inspection hints that the semantic incongruity alone elicits a larger N400 than the semantic violation in the presence of a codeswitch, especially over medial posterior sites. However, based on the difference waves, there was no difference between the semantic congruity effect in the presence of a codeswitch (M = −1.004 μV, SE = 0.147) compared to the effect in the absence of a codeswitch (M = −0.815 μV, SE = 0.154) [F_(1,23)_ = 0.720, *p* = 0.405]. The apparent visual difference stems from a more positive waveform for codeswitches (M = 1.981 μV, SE = 0.314) relative to the control (M = 1.384 μV, SE = 0.366), which trends towards significance during the N400 time window (*p* = 0.056).

There was no significant main effect of Code Switching [F_(1,23)_ = 3.820, *p* = 0.063], no interaction between Code Switching and Semantic Congruity [F_(1,23)_ = 0.725, *p* = 0.403], and no interaction between Semantic Congruity, Code Switching and Electrode [F_(4.3,98.7)_ = 1.334, *p* = 0.261], meaning that codeswitching did not affect semantic processing in this window. A significant interaction between Code Switching and Electrode [F_(3.9,88.9)_ = 3.727, *p* = 0.008] was found, so a distributional analysis was performed. An interaction between Code Switching and Laterality [F_(1,23)_ = 7.676, *p* = 0.011] indicated that responses to codeswitches were more positive than non-switches over medial sites [F_(1,23)_ = 5.260, *p* = 0.031], but not lateral sites [F_(1,23)_ = 1.339, *p* = 0.259]. An interaction between Code Switching and Anteriority indicated that codeswitches elicited a positivity compared to non-switches that was significant only over posterior sites [F_(1,23)_ = 11.529, *p* = 0.002]. Together this indicates that the Code Switching effect is maximal over medial posterior sites. No other interactions were significant.

### 3.2. Left Anterior Negativity (LAN) Region of Interest Analysis

**Figure 3.**
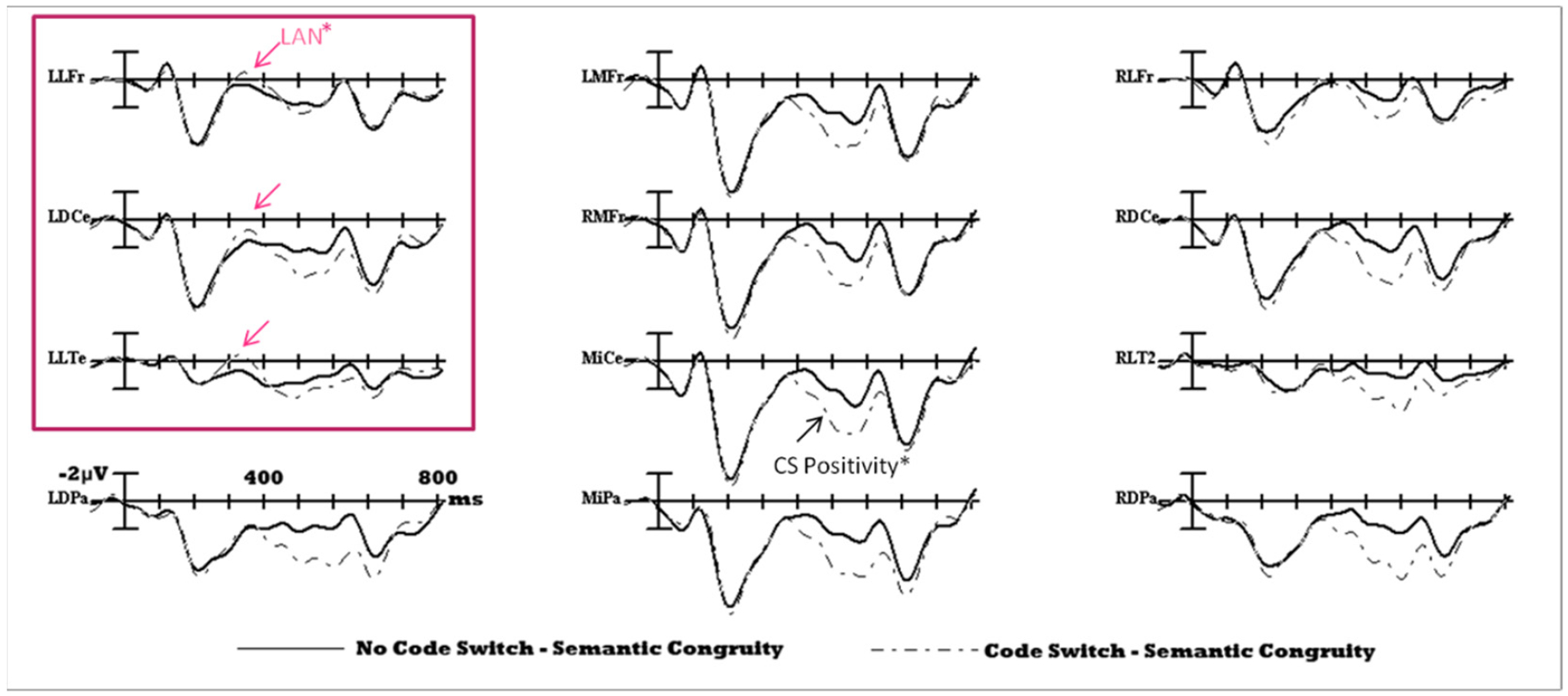
Electrophysiological correlates of codeswitching. A code-switching positivity can be observed from 320–650 ms post stimulus onset. A negativity is also observed over left anterior lateral sites (* *p* < 0.05).

A negativity over left lateral sites was visible, similar to the one observed for codeswitches in Moreno et al. (2002) [Figure 3]. Based on Moreno’s analysis, we performed a LAN region of interest analysis from 275–400 ms. A t-test was conducted to compare the mean amplitude of the left anterior electrodes (LLFr, LDCe, and LLTe) for the codeswitch and control condition. Responses to codeswitches were more negative in amplitude compared to non-switches [t_(23)_ = 2.248, *p* = 0.034].

### 3.3. LPCa Mean Amplitude 500–650 ms

In the LPCa window, a main effect of Code Switching was found [F_(1,23)_ = 10.403, *p* = 0.004], with codeswitches eliciting more positive amplitude than non-switches. A main effect of Electrode [F_(4.0,91.1)_ = 22.158, *p* < 0.001] and an interaction of Code Switching by Electrode [F_(3.6,83.6)_ = 12.034, *p* < 0.001] led to distributional analyses. Interactions of Code Switching with Hemisphere [F_(1,23)_ = 6.223, *p* = 0.020] and Code Switching with Laterality [F_(1,23)_ = 5.372, *p* = 0.030], and a three way interaction of Code Switching by Hemisphere by Laterality [F_(1,23)_ = 7.002, *p* = 0.014] indicated that the Code Switching Effect (greater positivity for switches than non-switches) was significant over both medial and lateral sites in both hemispheres, but most positive for medial sites over the right hemisphere. An interaction of Code Switching by Anteriority [F_(1.3,30.1)_ = 20.754, *p* < 0.001] indicated that the codeswitching effect was only significant over central and occipital sites and largest over occipital sites. Thus, the Code Switching effect is strongest over medial posterior sites in this time window and the LPCa window (500–650 ms) overlaps in time with the Code-Switching Positivity window (320–650 ms).

No main effect of Semantics was found in this time window [F_(1,23)_ = 0.053, *p* = 0821]. However, an interaction of Semantics by Electrode [F_(3.8,86.6)_ = 5.161, *p* = 0.183] led to distributional analyses. Interactions between Semantic Congruity and Anteriority [F_(1.3,30.6)_ = 10.559, *p* = 0.001] and Semantic Congruity, Laterality and Anteriority [F_(2.4,54.6)_ = 4.474, *p* = 0.012] revealed that semantic incongruities elicit a significantly larger positivity than semantic congruities at medial and lateral occipital sites [F_(1,23)_ = 5.081, *p* = 0.034; F_(1,23)_ = 13.750, *p* = 0.001, respectively] and lateral central sites [F_(1,23)_ = 5.203, *p* = 0.032]. While the Code Switching effect continued to be more positive for switches than non-switches, the Semantic effect reversed from the previous N400 window, with incongruous being now more positive than congruous words.

**Figure 4.**
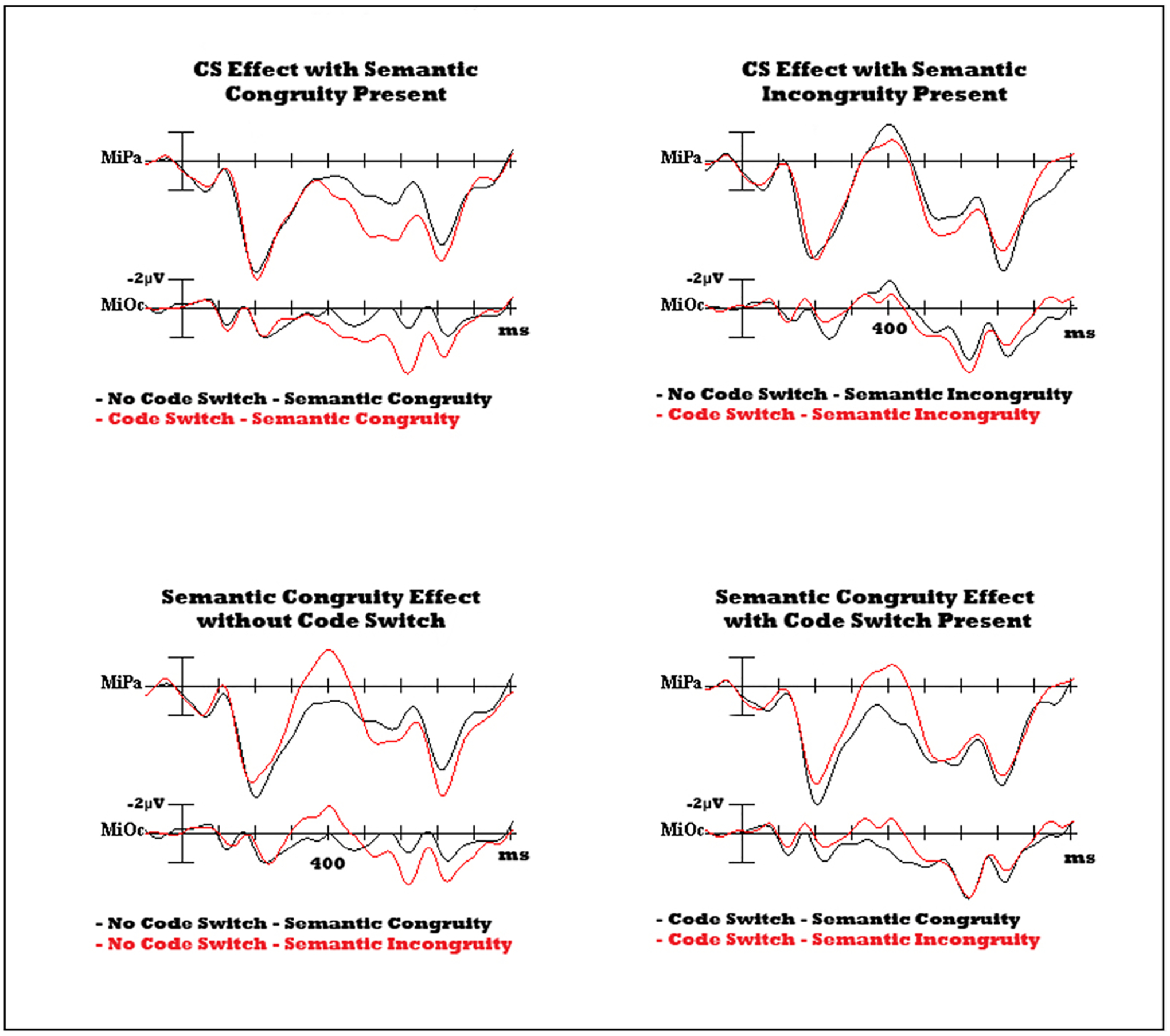
Interactions of code-switching and semantic congruity.

Interactions were also found between Code Switching by Semantics [F_(1,23)_ = 13.820, *p* = 0.001] and Code Switching by Semantics by Electrode [F_(4.2,97.4)_ = 2.567, *p* = 0.040]. Pairwise comparisons with Bonferroni correction revealed that the codeswitching effect (larger positivity for codeswitched versus non-switched words) was significant only in the absence of a semantic violation (*p* < 0.001), and not in the presence of a semantic violation (*p* = 0.541) [Figure 4]. Interactions of Code Switching by Semantics by Laterality [F_(1,23)_ = 13.158, *p* = 0.001] and a four-way interaction of Code Switching, Semantics, Laterality, and Anteriority [F_(2.4,55.5)_ = 3.550, *p* = 0.028] revealed that a widespread codeswitching effect is found in the absence of a semantic violation – largest over medial occipital sites and significant at all but lateral prefrontal sites. However, in the presence of a semantic incongruity, the effect is only significant at occipital sites, both lateral (*p* = 0.034) and medial (*p* = 0.028).

Conversely, the semantic congruity effect (larger positivity for semantic incongruities than congruities) was significant only when there was no codeswitch (*p* = 0.040). Interactions of Code Switching by Semantics by Laterality [F_(1,23)_ = 13.158, *p* = 0.001] and a four-way interaction of Code Switching, Semantics, Laterality, and Anteriority [F_(2.4,55.5)_ = 3.550, *p* = 0.028] revealed that in the absence of a codeswitch, the semantic effect is significant over central (*p*_medial_ = 0.023; *p*_lateral_ = 0.002) and occipital sites (*p*_medial_ = 0.002; *p*_lateral_ < 0.001), and largest over medial electrodes. In the presence of a codeswitch, there was a trend towards a reversal of this effect (semantic congruities are more positive than incongruities) (*p* = 0.066) [Figure 4], which became significant at frontal and prefrontal sites, and was largest over medial prefrontal sites (*p* = 0.013). No other interactions were significant.

### 3.4. LPCb Mean Amplitude 650–800 ms

No main effects of Semantics [F_(1,23)_ = 1.385, *p* = 0.251] or Code Switching [F_(1,23)_ = 0.304, *p* = 0.587] were found in this time window. However, an interaction of Semantics and Code Switching [F_(1,23)_ = 6.582, *p* = 0.017], along with subsequent pairwise comparisons, revealed that the Semantic effect (semantic incongruities are more positive than congruities) is significant in the absence of a codeswitch (*p* = 0.044), but not in the presence of one (*p* = 0.574). The Code Switch effect (switches are more positive than non-switches) was marginally significant (*p* = 0.065) in the absence of a semantic violation and not significant in the presence of one (*p* = 0.275). Main effects of Electrode [F_(3.5,79.5)_ = 15.942, *p* < 0.001] and interactions of Semantics with Electrode [F_(3.7,84.2)_ = 3.047, *p* = 0.025] and Code Switching by Semantics by Electrode [F_(3.5,80.5)_ = 3.306, *p* = 0.019] led to distributional analyses. Interactions of Code Switching by Semantics by Laterality [F_(1,23)_ = 9.700, *p* = 0.005] and Code Switching by Semantics by Anteriority [F_(1.3,30.8)_ = 4.602, *p* = 0.030] indicated that semantic incongruities are more positive than congruities only in the absence of a codeswitch and only over medial (*p* = 0.031) occipital sites (*p* < 0.001) and central sites (*p* = 0.003). The codeswitching effect is only significant in the absence of a semantic incongruity over lateral sites (*p* = 0.046), but not medial sites (*p* = 0.097). An interaction of Semantics by Anteriority [F_(1.3,29.5)_ = 5.544, *p* = 0.019] indicated that semantic incongruities elicited a positivity compared to congruities only over occipital sites (*p* = 0.003), with marginal significance over central sites (*p* = 0.061). No other theoretically relevant interactions were significant.

### 3.5. Code Switching Positivity 320–650 ms

In this expanded window, main effects of Code Switching [F_(1,23)_ = 9.249, *p* = 0.006] and Semantics [F_(1,23)_ = 27.919, *p* < 0.001] reached significance. Amplitudes to semantic incongruities were more negative than non-violations, and codeswitches were more positive than non-switches. A main effect of Electrode [F_(4.7,108.9)_ = 11.549, *p* < 0.001] and interactions of Electrode with Semantics [F_(3.5,80.0)_ = 4.433, *p* = 0.004] and Electrode with Code Switching [F_(4.5,103.9)_ = 7.752, *p* < 0.001] led to distributional analyses. The interaction of Code Switching by Semantics by Electrode was not significant.

An interaction of Code Switching by Anteriority [F_(1.3,29.5)_ = 12.402, *p* = 0.001] indicated that the Code Switching Effect was only significant over central and posterior sites, and maximal over posterior sites. Interactions of Code Switching and Laterality [F_(1,23)_ = 8.498, *p* = 0.008] and Code Switching and Hemisphere [F_(1,23)_ = 4.752, *p* = 0.040] indicated that the Code Switching Effect was significant over both medial and lateral sites and in both hemispheres, but larger over right medial sites.

An interaction of Semantics by Laterality [F_(1,23)_ = 22.643, *p* < 0.001] and Semantics by Laterality by Anteriority [F_(2.4,54.4)_ = 6.121, *p* = 0.002] indicated that semantic incongruities elicit a larger negativity compared to congruous words, and this effect was significant over all sites except lateral occipital sites. The negativity for semantic violations is largest over medio-central sites—consistent with the N400.

Finally, an interaction of Code Switching by Semantics [F_(1,23)_ = 5.316, *p* = 0.030] and Code Switching by Semantics by Laterality [F_(1,23)_ = 8.104, *p* = 0.009] indicated that the semantic incongruities elicit a negativity compared to congruities both in the presence (*p* < 0.001) and absence (*p* = 0.031) of a codeswitch, but the effect is largest in the presence of a codeswitch. In both cases, the semantic effect is larger over medial than lateral sites. The Code Switching effect is only significant in the absence of a semantic violation (*p* = 0.001) but not in the presence of a semantic violation (*p* = 0.455). It is largest over medial sites but significant over all sites. This interaction occurs in part due to overlap of this time window with both the N400 and the onset of the LPC to a semantic violation. No other theoretically relevant interactions were significant. The analysis in this larger window shows the effects of semantics on the N400 and the effects of codeswitching and semantics on the LPC, however, the more refined analysis of the smaller windows shows differences across the LPCa and LPCb windows.

### 3.6. Individual Differences

As in Moreno et al. (2002), we performed regression analyses to explore whether the main effects, N400 responses to semantic violations and LPC responses to codeswitches, were correlated with any of our language proficiency measures or frequency of daily codeswitching measured with ACSES.

#### 3.6.1. N400 (Semantic Congruity)

Regression analyses were performed on N400 amplitude of the semantic congruity effect (i.e., the averaged amplitude of all 26 electrodes in the semantic incongruity condition minus the control condition) and N400 peak latency using the following covariates: Woodcock RMI in English, Woodcock RMI in Spanish, and ACSES scores. There were no correlations of any factors with N400 mean amplitude. Spanish proficiency measured by the Woodcock RMI correlated negatively with N400 peak latency [r_(22)_ = −0.421, R^2^ = 0.177, F_(1,22)_ = 4.741, *p* = 0.040], indicating that the N400 effect is earlier in bilinguals with greater proficiency in the base language of the sentences, similar to Moreno et al. (2002). Semantic access may occur earlier in bilinguals with greater proficiency. There was no correlation between peak latency with English proficiency or with ACSES scores.

#### 3.6.2. LCPa and LCPb

No significant correlations were found for the LPCa or LPCb windows (measured separately) between Spanish language proficiency and the codeswitch effects of latency or amplitude (mean of all electrodes; codeswitch minus control condition) or for the semantic congruity effect (mean of all electrodes; semantic incongruity minus control). English RMI was positivity correlated with both the Code Switch effect [r_(22)_ = 0.478, R^2^ = 0.229, F_(1,22)_ = 6.520, *p* = 0.018] and the Semantic effect [r_(22)_ = 0.447, R^2^ = 0.200, F_(1,22)_ = 5.493, *p* = 0.029] during the LPCb. As English proficiency increased, the LPCb to both a codeswitch and a semantic violation increased in amplitude. No other correlations with proficiency were significant in these time windows.

**Figure 5.**
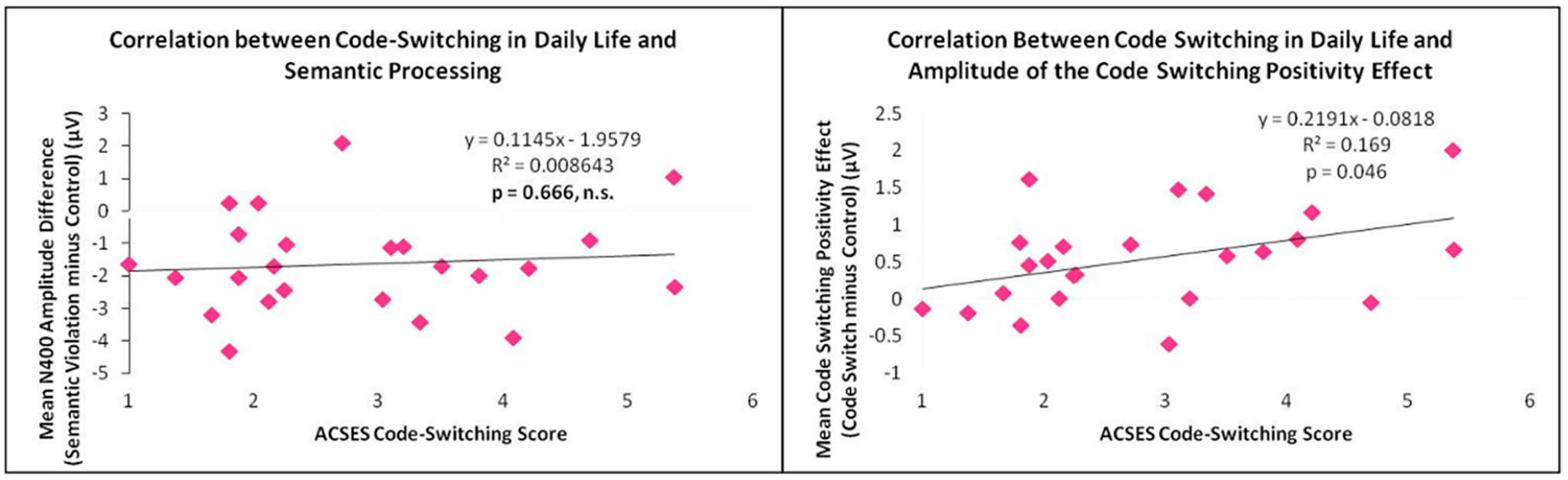
Correlations of frequency of code-switching with the amplitude semantic congruity effect on the N400 (**left panel**) and the amplitude of the code-switching positivity (320–650 ms; **right panel**).

ACSES scores were positively correlated with the amplitude of the Code Switch Effect, when measured from 320–650 ms [r_(22)_ = 0.411, R^2^ = 0.169, F_(1,22)_ = 4.480, *p* = 0.046]. As ACSES scores increased, i.e., as frequency of code-switching in daily life increased, mean amplitude of the codeswitching positivity also increased [Figure 5, right panel]. The correlation between ACSES and the Code Switch Effect was also measured during the LPCa, which partially overlaps with the codeswitching positivity, and LPCb windows. The correlation was significant during the LPCa from 500–650 ms [r_(22)_ = 0.415, R^2^ = 0.172, F_(1,22)_ = 4.582, *p* = 0.044] but not significant during the LPCb from 650–800 ms [r_(22)_ = 0.376, R^2^ = 0.141, F_(1,22)_ = 3.624, *p* = 0.070]. No correlation between ACSES and the Semantic Congruity Effect was found during these time windows [Figure 5, left panel]. ACSES scores were not correlated with the onset of the codeswitch positivity.

## 4. Discussion

We used ERPs to determine the time course of processing a codeswitch and how codeswitching interacts with access to the meaning of a word. We also investigated how codeswitching experience modulates comprehension and integration of a codeswitched word. Our data suggest that semantic level processing in a sentence context is not dependent on language identification in proficient bilinguals, and that increased frequency of codeswitching mainly affects the cost of processing a switch at post lexico-semantic integration.

As expected, a widely distributed, right medio-central N400 was observed in response to semantically implausible nouns (see Kutas and Federmeier 2011; Kutas and Hillyard 1980b). This was true regardless of whether the noun was in the same or different (codeswitch) language as the sentence. That is, a codeswitch did not modulate the amplitude of the N400, either for congruent or incongruent target words. The N400 was followed by a late positivity, typical of a semantic P600 (for a review, see Bornkessel-Schlesewsky and Schlesewsky 2008). In contrast, codeswitches elicited a LAN followed by a late positivity, with both effects overlapping in time with the N400, replicating prior findings (Moreno et al. 2002; Van Der Meij et al. 2011). There was an interaction between semantics and codeswitching only at the later part of the positivity (LPCb), but not at the LAN, N400 or LPCa. This interaction was sub-additive, such that codeswitches and semantic incongruities elicited equivalent LPC amplitude individually as did words that were both codeswitched and semantically incongruous. Language proficiency in this balanced bilingual population had two effects. Proficiency in Spanish (e.g., the matrix language) modulated N400 latency, but not N400 amplitude, while proficiency in English (codeswitch) modulated the semantic P600 and the codeswitching positivity. Finally, codeswitching habits modulated the late positivity, with larger positive amplitude for individuals who are more frequent codeswitchers. Codeswitching habits did not modulate the N400. We discuss the significance of each of these findings below.

### 4.1. Lack of a Codeswitch Effect on the N400 Indicates That Access to Meaning Is Independent of Language Membership

Ng and Wicha (2013) found that in single word recognition, meaning is accessed prior to language membership during lexical selection. In their study, while reading sequentially presented words in English and Spanish, bilinguals were asked to press a button each time they saw a word from a specific semantic category (i.e., people words), but only if they appeared in a specific language. They found that bilinguals started to categorize the words as targets and non-targets before rejecting the words from the non-target language. This happened especially when the non-target language was the more frequently used language (English), making it harder to ignore. Their findings implied that early proficient bilinguals can prioritize the meaning of words independent of language membership (for an example of when language membership is prioritized see Hoversten et al. 2015).

Similarly, in the current study, there was no effect of language membership, namely the language in which the target word was presented, and there was no interaction of codeswitching and semantic congruence on the amplitude or latency of the N400. Codeswitched words were processed in the same time course, eliciting N400 modulations in the same latency around 300 to 500 ms, and to the same degree, eliciting indistinguishable amplitude modulations, as non-switched words. As with single word processing (Ng and Wicha 2013), this suggests that early proficient bilinguals can prioritize meaning over the language of the presented word during this early stage of comprehension indexed by the N400, at least when the sentence context supports a specific continuation. This finding is also in line with Valdés Kroff et al. (2020) where proficient bilinguals showed no modulation of the N400 to codeswitches in a similar design, and with Moreno et al. (2002) who suggested that the cost of processing a codeswitch may not occur at the semantic level and instead at a later stage reflected by a late positivity.

These findings differ, however, from studies that do show a modulation on the N400 (Table 1). Proverbio et al. (2004) reported an enhanced N400 for semantically acceptable codeswitches compared to sentences without language switches. Proverbio et al. used a blocked presentation and included multilingual professional interpreters, which could complicate generalization of their findings. Nevertheless, this was not the only study to show a codeswitching modulation on the N400. Several studies have shown an asymmetric effect of codeswitching, where switching into the non-dominant language elicits a modulation of the N400 (Liao and Chan 2016). Others have reported switch effects in both directions in habitual switchers (Yacovone et al. 2021), or even in language learners (Ruigendijk et al. 2016). Often in these studies language proficiency was measured by self-assessment, where as our population was carefully assessed for proficiency in both languages. So, perhaps it is language proficiency that affects access to the meaning of words rather than codeswitching, per se.

Some studies have suggested that switching frequency might modulate the N400, with studies showing an N400 modulation in habitual switchers (Litcofsky and Van Hell 2017) and others suggesting the opposite (Gosselin and Sabourin 2021). Using the reliable and validated ACSES survey to measure self-reported switching habits, we did not observe an effect of switching frequency on the amplitude or latency of the N400. This again suggests that differences in how proficiency is measured may contribute to when a modulation of the N400 is observed, rather than switching habits, per se.

In fact, we observed a modulation of the N400 with proficiency, partially replicating findings from Moreno et al. (2002). Increased proficiency in the language of sentence presentation (Spanish) led to earlier N400 latency. This N400 latency shift even with small proficiency differences in our more balanced bilingual population indicates that N400 *latency* is quite sensitive to language proficiency. Semantic access may occur earlier in bilinguals with greater proficiency.

In brief, codeswitching did not modulate the N400 in this study, revealing that at least for early fluent bilinguals, code-switches are not processed as semantic violations and the brain can prioritize the meaning of words regardless of which language they appear in during sentence reading.

### 4.2. Codeswitching LAN—A Different Kind of Negativity

Encountering a codeswitched word elicited a LAN, a negativity that overlaps in time with the N400, but has a different distribution and is commonly observed to codeswitched words both in a sentence context and isolated word pairs (Barber and Carreiras 2005). A LAN has also been observed in response to syntactic agreement violations and has been attributed to early detection of a mismatch between morphosyntactic features (and difficulty integrating these features into the syntactic structure; Friederici 2002; Münte et al. 1997a, 1997b), or to the taxing of working memory (Kluender and Kutas 1993). The sentences in the current study were presented in Spanish, a language with rich morphological marking for gender and number agreement. Therefore, it is possible that the mismatch between the Spanish article and the English word contributed to the LAN.

Similarly, Litcofsky and Van Hell (2017) observed a small LAN on the second codeswitched word in a sentence that began in Spanish and switched the remainder of the sentence into English. In line with this morphological mismatch hypothesis, they did not observe this LAN for switches out of English into Spanish. However, a LAN has also been observed for codeswitches embedded in English sentences, a language with poor morphological markings (Moreno et al. 2002). Because the LAN is also linked to working memory load (Kluender and Kutas 1993), Moreno et al. (2002) suggested that codeswitches incur a working memory cost, perhaps in order to integrate morphological cues (such as agreement) across languages. Thus, the LAN in the current study likely reflects this cross-language syntactic integration, while the N400 reflects initial access to semantic memory.

### 4.3. Overlap of the Codeswitching Positivity with the N400 Suggests Parallel Processing of Meaning and Language Membership

The temporal overlap between the N400 and codeswitching positivity suggests parallel processes. These findings, along with Ng and Wicha (2013) in single words, provide evidence that language membership does not need to be identified prior to semantic retrieval, but rather these are separable processes that can occur in parallel (although for examples where it does not occur in this order, see Hoversten and Traxler 2020; Hoversten et al. 2015, 2017).

Early models of word recognition proposed a serial word recognition system in which language membership was identified prior to lexico-semantic retrieval (Kirsner et al. 1984). Our data challenges a serial model in favor of one which allows for parallel processing of semantics and language membership. Our results are more consistent with the BIA+ model of word recognition, in which access to semantics occurs independently of identification of the language (Dijkstra and Van Heuven 2002). In BIA+, identification of language membership occurs through language nodes, or labels, which provide information about properties of the word, but do not substantially affect word activation and semantic retrieval (Van Heuven and Dijkstra 2010). According to the model, word recognition is language non-selective, meaning that potential word choices in both languages are activated (Dijkstra and Van Heuven 2002).

Note that the need for an explicit language node may not be obligatory and may be instead an emergent property of the lexical spread within the lexicon (e.g., Elman 1993). Nevertheless, our results suggest that knowledge of the language that the word belongs to is not necessary for initial access of semantic level information, as indexed by the N400. Gullifer et al. (2013) showed that being in a codeswitching context versus a single language context does not change the non-selective nature of language comprehension during sentence reading. We have furthered this finding by showing that semantic access during sentence reading is unaffected by encountering a codeswitch. These data indicate that access to semantics occurs independently of and sometimes even prior to accessing language membership, at least for early balanced bilingual adults.

### 4.4. P600, LCP and Other Positivities

There has been a confluence of late positive modulations reported in the sentence comprehension literature. These positivities have been observed in response to a wide variety of stimuli, including semantic violations (Chaire 2013; Wicha et al. 2004), syntactic or morphosyntactic-agreement violations (Barber and Carreiras 2005; Osterhout and Holcomb 1992; Osterhout and Mobley 1995), codeswitches (Moreno et al. 2002), and changes in form (Kutas and Hillyard 1980a). Importantly, these late positivities are also observed in the absence of an overt violation of meaning or grammar and may instead reflect continued processing of unexpected stimuli (Leckey and Federmeier 2020; Van Petten and Luka 2012). Because of the significant overlap in time, occurring around 500–900 ms after stimulus onset, and distribution over posterior scalp electrodes, it has been difficult to determine if these positivities are neurologically and functionally distinct or similar events. In turn, these late positivities are often not treated separately in the literature. However, there have been recent attempts to distinguish them (Leckey and Federmeier 2020; Van Petten and Luka 2012).

The P600 was first described as a brain response specific to syntactic violations (Osterhout and Holcomb 1992; Hagoort et al. 1993) but has since been observed in response to non-syntactic, even non-linguistic, stimuli and does not even require an overt violation (Van Petten and Luka 2012). Nevertheless, although the P600 may not be a response specific to grammar violations, encountering syntactic difficulties in a sentence context (but not in isolated word pairs; Barber and Carreiras 2005) reliably elicits this P600 response. One interpretation of this syntactic P600 is that it reflects revisiting, reanalysis or reprocessing after an anomaly has been detected (Vaughan-Evans et al. 2020; Leckey and Federmeier 2020; Kuperberg 2007; Van Petten and Luka 2012; Brouwer et al. 2017), for integration of the word within the broader sentence context (Salmon and Pratt 2002). Importantly, given that the syntactic P600 is sensitive to stimulus features, such as task relevance (e.g., making grammaticality judgments) and probability (Coulson et al. 1998b, cf Osterhout and Mobley 1995), it has been argued that this ERP component may be related to the well-studied domain-general P300 (specifically the P3b), which is associated with context or memory updating (Coulson et al. 1998a, 1998b; Donchin 1981; Verleger 1988; Gunter et al. 1997; Osterhout and Mobley 1995; Van Petten and Luka 2012).

Semantic anomalies can also elicit a late positivity, which often but not always follows the N400 (Kuperberg 2007; Van Petten and Luka 2012). This positivity, referred to as the semantic P600, post-N400 positivity or late positive component (LPC) (Leckey and Federmeier 2020; Van Petten and Luka 2012), appears in response to incongruous, overt violations of meaning, like the ones used in this study, and also to less overt manipulations, such as thematic role violations (e.g., “For breakfast, the eggs would only eat toast and jam”; Kuperberg et al. 2003). The semantic P600 does not tend to appear for sentence continuations that are unexpected but semantically plausible^4^ and has been explained by similar processes as the syntactic P600, reflecting reprocessing or reanalysis after encountering a violation of meaning (Kuperberg 2007). Recent evidence in older adults, however, suggests that the semantic P600 may be separable from the P600 driven by syntactic errors (Leckey and Federmeier 2020). For example, the P600 can begin earlier for syntactic errors than to semantic incongruities and has even been described as having two separate phases, with an earlier one sensitive to syntactic information and the later one sensitive to both syntax and semantics (Wicha et al. 2004; Guajardo and Wicha 2014; Barber and Carreiras 2005).

The codeswitching positivity overlaps in time and distribution with the semantic and syntactic P600s (see Table 1) (Litcofsky and Van Hell 2017; Moreno et al. 2002; Van Der Meij et al. 2011). This codeswitching positivity has also been associated with sentence reanalysis, similar to the P600 (Litcofsky and Van Hell 2017), or as indexing the unexpected nature of the switched word (Moreno et al. 2002). However, it is unclear if and how the codeswitching positivity commonly elicited to language switches in a sentence context relates to either of these P600 responses. We explore below how our data informs what kind of effect this codeswitching positivity might be.

### 4.5. The Codeswitching Positivity vs. the Semantic P600

Semantic incongruities in the current study elicited a typical semantic P600 from 500–800 ms post-stimulus onset, consistent with previous findings where overt semantic incongruities are presented (Chaire 2013; Wicha et al. 2004). Codeswitched nouns also elicited a late positivity that started earlier than the semantic P600, but overlapped with the semantic P600 in distribution, maximal over medial posterior electrodes. Critically, semantic violations that were also codeswitched did not elicit any additional modulation of the LPC/semantic P600 (Figure 4), indicating that these positivities are not additive. The larger amplitude for the semantic P600 than the codeswitch positivity may indicate that reprocessing is more difficult for meaning violations than for language switches. The ease of integrating a codeswitch is in line with studies showing that the processing cost for a codeswitch can be eliminated under natural circumstances and meaning is obtained without delay for codeswitches (Chan et al. 1983; Gullifer et al. 2013). In theory, this would be facilitated if words from both languages were activated during word recognition (Caramazza and Brones 1979; Dijkstra and Van Heuven 2002), which has previously been demonstrated even in single language sentence contexts (Duyck et al. 2007; Schwartz and Kroll 2006; Titone et al. 2011; Van Assche et al. 2011; Van Assche et al. 2012). Words from both languages may be activated and considered within the sentence context – resulting in faster integration of codeswitches than semantic violations.

Does this mean that the codeswitching LPC and the semantic P600 are the same positivity? This is not clear. We found that proficiency in the language of the code-switched word (English) modulated the amplitude of the semantic P600 as well as the codeswitching positivity, specifically the later LPCb but not the earlier LPCa. As proficiency in English (codeswitch) increased, the amplitude of the positivity to both a codeswitch or a semantic incongruity increased. This might suggest that this later positivity relates to similar sentence reprocessing for both semantic anomalies and unexpected codeswitches. Yet, the direction of this modulation is curious. One might expect that better proficiency in a language would elicit easier reprocessing and therefore reduced positivity, opposite of what we observed. In fact, both Moreno et al. (2002) and Litcofsky and Van Hell (2017) observed exactly this. As proficiency in the switched language increased the codeswitching positivity to the target word decreased.^5^ Similarly, Ruigendijk et al. also observed larger codeswitching positivity in less proficient learners of a language (Ruigendijk et al. 2016), although Liao and Chan (2016) observed a codeswitching positivity in both directions for bilingual switching into their more proficiency and into their less proficient languages.

Litcofsky and Van Hell (2017) measured the effect of proficiency in a highly fluent, but less balanced Spanish–English population, most of whom codeswitched habitually. They observed an asymmetrical effect, with a codeswitching positivity only when switching into the weaker language. They inferred that the weaker language may entail more effortful processing and sentence-level restructuring after encountering a switch. This interpretation was corroborated by EEG analysis in the time-frequency domain, which showed a decrease in lower beta band oscillations (15–18 Hz) only when switching into the weaker language. Decreases in the lower beta band have been observed to sentence-level processing, such as in response to syntactic violations. In contrast, they observed power increases in theta band oscillations (4–7 Hz), related to lexico-semantic processing and lexical inhibition, when switching into the dominant language. They suggested that switching into a lower proficiency language might require effortful sentence-level restructuring and shifting between languages, while switching into the dominant language results in a release of L1 inhibition at the lexical-semantic level that was previously required to process sentences in the weaker language (Litcofsky and Van Hell 2017; Van Hell et al. 2018).

These inconsistencies in the effect of language proficiency across studies is puzzling. Given that our sample was balanced in proficiency across their languages, it is possible that the effects of proficiency in our data are tenuously linked to small fluctuations in proficiency. However, a different proficiency effect, the reduction in N400 latency with increased proficiency, is consistent with previous findings. Perhaps instead our proficiency effect reflects suppression of a stronger language; greater suppression would be needed as proficiency increases, eliciting larger LPC amplitude as an index of access to this inhibited language.

### 4.6. What Is the Codeswitching Positivity?

The behavioral literature shows a processing cost for reading a codeswitch (Macnamara and Kushnir 1971). This delay may be reflected in the codeswitching positivity. What is the codeswitching positivity?

Some clues can be gleaned from outside the language literature. It has been suggested that the codeswitching positivity may reflect task-set reconfiguration (Moreno et al. 2008). During non-language task switching, a sustained differential positivity (D-Pos) peaks approximately 300 to 400 ms following a cue to switch between two tasks relative to a cue not to switch (Karayanidis et al. 2003; Nicholson et al. 2005). This differential positivity is maximal over central-parietal sites, has been localized to the superior parietal cortex, and is associated with activating task rules during reconfiguration to a new task (Jamadar et al. 2009). D-Pos is followed by activation in the dorsolateral prefrontal cortex, a region postulated to initiate a cortical-subcortical loop involved in switching between both languages and non-language tasks (Abutalebi and Green 2008). A similar component to D-Pos for codeswitches compared to non-switches has been identified during language production (Blackburn 2013). However, activity in the dorsolateral prefrontal cortex following D-Pos might play a larger role during production, as it is not observed during comprehension of a codeswitch, at least not in the absence of a processing cost when reading short two-word sentences with switches (Phillips and Pylkkanen 2019).

The code-switching positivity observed here is similar to D-Pos in time-course and distribution and may reflect a language reconfiguration process in order to switch between languages. We suggest based on evidence that motor representations are modeled during auditory comprehension (Bangert et al. 2006), that the code-switching positivity may reflect activation of the motor representation for switching between languages. Two distinct processing streams have been implicated in auditory processing: a ventral pathway involved in mapping input to meaning and a dorsal sensorimotor pathway that enables mapping of the input to articulatory motor representations (see also Friederici and Gierhan 2013; Hickok and Poeppel 2007; Poeppel et al. 2012). It is possible that semantic processing occurs via the ventral stream and is reflected by the N400 and subsequent semantic P600. In contrast, codeswitch processing may occur along the dorsal pathway, resulting in a codeswitching positivity that reflects mapping of the input to the motor output necessary to articulate a switch into another language.

To test this prediction, factors known to increase D-Pos and/or articulation difficulty, such as stimuli that evoke both tasks (e.g., false cognates or words with similar cross-language orthography but different pronunciation) can be manipulated in a bilingual sentence reading paradigm. Ongoing research is investigating how code-switching experience modulates both D-Pos and the codeswitching positivity, to determine if these components are functionally similar.

Another possibility is that the codeswitching positivity is similar to a P600 or LPCa elicited in response to a syntactic violation (Hagoort et al. 1993; Osterhout and Holcomb 1992; cf. Van Der Meij et al. 2011). Codeswitches elicit a pattern similar to the LAN-P600 pattern commonly obtained for syntactic violations (Barber and Carreiras 2005; Osterhout and Mobley 1995). Similarly, processing sentential codeswitches has elicited neural oscillations at a frequency comparable to those elicited to syntactic violations (Litcofsky and Van Hell 2017). It has been suggested that the LPC may in fact represent two processes with two separate generators: a syntactic early phase and a later integration phase (Barber and Carreiras 2005). The differential positivity for codeswitches versus non-switches was only significant during the first half of the LPC. Although codeswitches do not necessarily create syntactic anomalies, perhaps the codeswitches are processed initially as syntactic anomalies. This explanation may be in line with the three processing steps proposed by Friederici and Kotz (2003): an initial structure building phase reflected by the LAN elicited to a codeswitch, semantic integration reflected by the N400, and a late syntactic integration phase reflected by the P600/LPC.

Finally, it has been suggested that both the semantic P600 and the codeswitching positivity are variants of a late P3 component (Van Der Meij et al. 2011; but see Leckey and Federmeier 2020 who argue that the semantic and syntactic effects are not the same). The LPC is often interpreted as a P3 which increases in amplitude for anomalous stimuli (Coulson et al. 1998b). Nieuwenhuis et al. (2005) proposed that late positivities such as the P3 may reflect phasic activity of the locus coeruleus-noradrenergic system, which works to increase sensitivity of neurons to incoming stimuli and to focus attention (Nieuwenhuis et al. 2005). Locus coeruleus activity is driven by task-relevant decision-making processes, i.e., identification of task-relevant stimuli and mapping these to appropriate responses, and is typically amplified in response to stimuli that are relevant to the current task.

According to this hypothesis, individuals have an internal model of the external world or current task structure, e.g., reading sentences in Spanish. When the current expectations are violated by a stimulus that does not match the task, e.g., an English word or a word that violates semantic expectations built by the sentence context, the locus coeruleus responds with phasic firing and a large P3 results. Increased locus coeruleus phasic firing facilitates responding to the outcome of the decision making process and results in less distraction by irrelevant stimuli and better performance on the task (Nieuwenhuis et al. 2005).

The positivities observed to codeswitches and semantic anomalies may reflect the brain activity evoked by the need to update the context in light of an unexpected word and may reflect both attention and decision making processes associated with this context updating (Polich 2007; Van Der Meij et al. 2011). If this is the case, both the codeswitching positivity and the semantic P600 may involve activation of a general anomaly detector and all or part of these positivities may involve the same generator (Barber and Carreiras 2005; Coulson et al. 1998b; Van Der Meij et al. 2011). This possibility is not entirely different from the task reconfiguration account described above, as the P3 is sensitive to task relevance (Coulson et al. 1998a) and D-Pos may also be a variant of the P3.

### 4.7. Code Switching Experience Modulates the Code Switching Positivity

Previous studies have found that increased code-switching experience in daily life reduces the response time cost of producing a code-switch (Prior and Gollan 2011). Of equal importance is the fact that codeswitches may be unexpected by the interlocutor and are known to incur a cost in comprehension time. A major question is whether this processing cost is attenuated with code-switching experience. We tested whether individual differences in codeswitching behavior tune neural sensitivity to processing a sentential codeswitch.

A correlation between how frequently an individual codeswitches and the amplitude of the codeswitching positivity was observed. Specifically, increased switching also increased the amplitude of the codeswitch positivity, suggesting that bilinguals who codeswitch more often in daily life may dedicate more resources to processing codeswitches. In contrast, there was no correlation between code-switching experience and semantic retrieval during the N400. This effect of codeswitching experience was specific to processing the language switch itself.

A larger codeswitching positivity for individuals who are more experienced with codeswitching may appear counterintuitive given findings that expertise often results in more efficient processing (e.g., during motor learning, see Landau and D’Esposito 2006). However, both the interpretation that the codeswitching positivity may be a form of a P3 or P600 and that it may reflect a task-decision stage of processing, leads to a prediction that the positivity would be positively correlated with experience. First, the P600 amplitude is positively correlated with proficiency and behavioral sensitivity to syntactic anomalies (McLaughlin et al. 2010). Thus, code-switching experience may increase sensitivity to a codeswitch. Second, the P3 amplitude is linked to attention control and task performance and is susceptible to both developmental change and individual differences (Johnstone et al. 1996; see Kok 2001 for a review; Ladish and Polich 1989). Larger P3 amplitudes are linked to more accurate and faster responses (Nieuwenhuis et al. 2011), and the P3 amplitude is larger in children with better capacity allocation—the ability to invest resources in a task (Jonkman et al. 2000). Wright et al. (2013) found that the P3 amplitude is larger during task performance for individuals who are experts at performing that task. They concluded that experts may engage selective attention more effectively during the task. Thus, if the codeswitching positivity is related to the P3 family of positivities, then this increase in the amplitude with more experience/expertise with codeswitching would be consistent with what is observed for P3 amplitude.

Similarly, individuals with experience codeswitching may be more inclined to allocate mental capacity (resources) into processing a codeswitch and may be more attentive to codeswitches than those less likely to encounter codeswitching in their daily lives. As discussed above, the enhanced P3 in daily codeswitchers may reflect an increased phasic locus coeruleus response which serves to focus attention and facilitate decision making processes (e.g., integration of the word into the sentence). The codeswitching positivity has also been likened to the positivity elicited during task-set reconfiguration, D-Pos (Moreno et al. 2008). We are currently testing the prediction that as code-switching experience increases, D-Pos elicited during both language and non-language task switching will also increase. In addition, these findings open the door to further studies linking this neurocognitive research paradigm with sociolinguistic research tradition. For instance, we are interested in testing differences in the code-switching positivity for habitual codeswitchers when they encounter a contextually predictable codeswitch.

It should be noted that we operationalized codeswitching frequency using the ACSES survey, which measures switching within conversations (i.e., being in a dense codeswitching context). Bilinguals were not categorized according to their language environment, as has been the custom in other studies (for a review, see Blackburn 2018). Thus, we were not able to distinguish between bilinguals who refrain from switching because they spend time in environments where only one language is appropriate (single-language context) and those who switch frequently across conversations but not within conversations (dual-language switching) (Green and Abutalebi 2013). Hofweber et al. (2016) suggested that bilinguals who spend time in a dense codeswitching context should exhibit increased monitoring skills and control when processing language switches, as a result of often having to be ready to anticipate a language switch. Further research is necessary to determine if the codeswitching positivity differs for bilinguals who spend time in a dual-language context when both languages are appropriate vs. those who spend time in a single-language context, or according to the specific type of codeswitching bilinguals encounter. Nonetheless, the findings herein validate ACSES and show that this bilingual phenomenon is independent of other measures, such as language proficiency.

## 5. Conclusions

In conclusion, processing of a codeswitch does not appear to interfere with semantic retrieval. Rather, in support of models such as BIA+, our findings indicate that identification of language membership and semantic retrieval occur in parallel and likely involve separate generators. A codeswitching positivity, which overlaps in time with the N400 to a semantic violation, was observed following a LAN to switches compared to nonswitches. A late positivity with similar distribution and overlapping time course to the codeswitching positivity was observed for semantic incongruities relative to congruities. The co-occurrence of a codeswitch and semantic violation resulted in a positivity that did not differ substantially from the positivity elicited to each individually. It is possible that these positivities reflect a general P3-like anomaly detector and reprocessing due to the unexpected event. Alternatively, the codeswitch positivity may reflect reconfiguration of the language at this later reprocessing stage, or less likely, the treatment of a code-switch as a syntactic violation in line with a syntactic P600. Codeswitching experience was found to modulate the codeswitching positivity, much like expertise and increased attention have been found to increase the amplitude of the P3 component. While further investigation is needed to elucidate the generators of the codeswitching positivity and the semantic P600, it is clear that the codeswitching positivity reflects a process distinct from and parallel to semantic retrieval, and that this process is affected by experience with codeswitching in daily life.

## Figures and Tables

**Table 1.** Summary of studies to date measuring ERPs to codeswitches during sentence comprehension. Key population and task factors are noted, including switching habits where reported, direction of switch where appropriate and format of sentences (written or auditory). Effects on N400, LAN and LPC components are specific to codeswitches; other relevant effects are noted, but additional effects may be reported in original studies.

Study	Population	Task	N400	LAN	LPC	Other
Moreno et al. 2002	English-Spanish; English dominant	Read English sentences; expected, lexical switch or Spanish (non-dominant) translation of expected word	No	Yes	Yes; modulated by Spanish proficiency earlier and smaller for more proficient	Late Frontal positivity for lexical and code switches
Proverbio et al. 2004	Multilingual interpreters	Read English and Italian sentences; blocked by mixed (codeswitch) and unmixed sentences but mixed by matrix language	Larger N400 overall for mixed (switched) than unmixed	Not reported	Not reported	Report a modulation of the N1 for codeswitches
Van Der Meij et al. 2011	Late learners of English	Read English sentences (L2) switch into L1 Spanish (dominant); targets were adjectives	Small but sig; larger for high English proficiency	Yes	Yes	Frontal positivity to CS starts earlier than posterior LPC; report ortho N250 that might be N400
Ng et al. 2014	Balanced early Spanish–English	Read English stories with embedded Spanish nouns and verbs	Reduced for switches over frontal sites but not medial central (more focal)	Yes	Yes; earlier for nouns than verbs (LPCa)	No effect of story position on codeswitch effects
Liao and Chan 2016	Mandarin-Taiwanese early bilinguals; dominant Mandarin	Spoken sentences in both languages	Only when switching into non-dominant language	Only when switching into non-dominant language	Yes in both directions	
Ruigendijk et al. 2016	Russian learners of German; intermediate and high proficiency & native German	Spoken German sentences with Russian codeswitch or German semantic violation	Yes, even for monolinguals	Not reported	Yes, larger in less proficient learners	Reported N400 possibly N2 modulations
Litcofsky and Van Hell 2017	L1 Spanish, early L2 English; Dominant L2; habitual codeswitchers	Written sentences English and Spanish translations; multi-word switch after target noun	No	No	Only when switching into non-dominant language	
Fernandez et al. 2019	L1 Spanish, early L2 English; $\frac{1}{2}$ Dominant L2; equal daily use of both; habitual codeswitchers	Auditory sentences English and Spanish with multiword codeswitch after target noun	Yes; in both switch directions	No	Only when switching into non-dominant language	
Vaughan-Evans et al. 2020	Welsh-English; proficient in both	Read English and Welsh matrix language; manipulated adjective-noun word order as per English or Welsh; codeswitch on every trial; semantic acceptability judgment	No	Yes, for both switch types	LPC only for matrix language framework switches	
Kaan et al. 2020	Spanish–English, early, English dominant	Read English sentences with multiword Spanish switch (non-dominant) starting with function word; presence of bilingual or monolingual confederate	No; possibly because switch measured at function word	No	Yes; reduced with presence of bilingual confederate	Early positivity
Valdés Kroff et al. 2020	Spanish–English early proficient; frequent switchers	Reading Spanish with English switched nouns with high/low expectancy at noun 2 × 2 w/switches	No	No	Yes	Early posterior positivity (P300?) modulated by switch frequency
Gosselin and Sabourin 2021	French-English, early and proficient; habitual and non-habitual switchers	Determiner phrase switches	Only for non-habitual switchers	Only for non-habitual switchers	Only for habitual switchers	LPC might be obscured by N400 in non-habitual group
Yacovone et al. 2021	Spanish–English; English dominant; frequent codeswitchers	Spoken English stories, high/low expectancy at target noun w/Spanish (ND) switches	Yes; non-dominant switch	No	Yes, regardless of expectancy	Same modulation for codeswitch, low expectancy non-switch and low expectancy switch
This Study	Spanish–English early and balanced proficiency; vary in switching habits	Spanish sentences with Single English word switch 2 × 2 w/semantic violations	No; only main effect of congruency	Yes	Yes; reduced with more switch frequency	

## Data Availability

The data presented in this study are available on request from the corresponding author.

## Footnotes

1 We present our research as it fits in the current body of literature. However, it should be noted that our original hypotheses were proposed and the research was conducted prior to the literature presented in Table 1 (Blackburn 2013).

2 Semantic violations with a codeswitch did not elicit an LPC in every electrode. Only electrodes in which a positivity was elicited were included in the onset calculation. N.B. Due to local maxima/minima in individual data, onset latencies were calculated on the grand averaged difference waves. Therefore, these values do not contain individual subject variance and do not allow for statistical comparisons.

3 We refer to the LPCa and LPCb elicited in response to a semantic violation here as the semantic positivity.

4 Failed semantic predictions have been reported to elicit a more frontally distributed positivity (Federmeier 2007).

5 Van Der Meij et al. (2011) found that as proficiency in the language of the sentence context increased the positivity in response to a codeswitched word also increased.
